# Building on the Doha Declaration: a white paper on advancing lifestyle medicine for non-communicable disease prevention and health system transformation

**DOI:** 10.3389/fpubh.2026.1873047

**Published:** 2026-07-07

**Authors:** Sohaila Cheema, Amit Abraham, Amy Mechley, Agnese Lapsa-Lešinske, Emanuela Mercore Huțanu, Jenny Sunghyun Lee, Sandra Daniela Lanza Sagardia, Shagufta Feroz, Sivaneswaran Poobalasingam, Ravinder Mamtani

**Affiliations:** 1 Institute for Population Health, Weill Cornell Medicine-Qatar, Doha, Qatar; 2 The American Board of Lifestyle Medicine and the International Board of Lifestyle Medicine, Temescal Valley, CA, United States; 3 Lifestyle Medicine Global Alliance, Vilnius, Lithuania; 4 Romanian Society of Lifestyle Medicine, Iași, Romania; 5 Korean College of Lifestyle Medicine, Seoul, Republic of Korea; 6 Chilean Society of Lifestyle Medicine (Sociedad Chilena de Medicina del Estilo De Vida), Universidad Andrés Bello, Concón, Chile; 7 Pakistan Association of Lifestyle Medicine, Riphah International University Islamic International Medical College, Islamabad, Pakistan; 8 Malaysian Society of Lifestyle Medicine, Kuala Lumpur, Malaysia

**Keywords:** chronic disease management, lifestyle medicine, non communicable disease, population health, prevention

## Abstract

**Introduction:**

Lifestyle Medicine (LM) is an evidence-based discipline focused on preventing, managing, and reversing non-communicable diseases (NCDs) through the adoption of healthy lifestyle behaviors. Recognizing its potential to reshape clinical practice and inform public health policy, the Doha Declaration on LM advocates for urgent and systematic integration of LM into healthcare systems worldwide.

**Methods:**

A consensus meeting was held by an expert panel of 26 global LM professionals, the findings and recommendations of which are documented in this white paper.

**Results:**

The white paper explores the implications of LM for global health outcomes, economic sustainability, and population well-being. It highlights the strategic importance of including LM into medical education, clinical guidelines and health governance frameworks. Furthermore, it identifies key challenges to implementation and proposes actionable solutions to advance LM as a cornerstone of equitable and sustainable healthcare delivery worldwide.

**Conclusion:**

This white paper, based on the recommendations of the Doha Declaration on LM, demonstrates that LM can reduce the growing burden of NCDs. However, for LM to make a meaningful impact, it needs to be incorporated into healthcare systems and educational programs, and be supported by policies that align with local cultures, resources, and practices. These combined efforts can help address unhealthy behaviors, and the underlying factors that influence disease risk and health outcomes.

## 1 Introduction

Lifestyle Medicine (LM) has emerged as a critical approach to the prevention and management of Non-Communicable Diseases (NCDs), supported by growing evidence of its effectiveness in improving health outcomes and extending longevity (1). Unlike conventional approaches that often prioritize pharmacological or surgical interventions, LM emphasizes the unhealthy behaviors that drive NCD risk. LM interventions are grounded in six foundational pillars: (i) a healthy, whole-food predominant plant-based eating pattern, (ii) physical activity, (iii) stress management, (iv) restorative sleep, (v) connectedness, and (vi) avoidance of risky substances (2). By targeting these unhealthy behaviors, LM can address the root causes of many NCDs. This approach aligns with global public health priorities, which advocate for preventive strategies and population-level interventions that reduce chronic NCD burden and healthcare expenditures (3). Integrating LM into clinical guidelines and health systems therefore represents a strategic and evidence-based response addressing the escalating prevalence of NCDs.

Recognizing the potential of LM to address NCDs, a global symposium was convened in February 2024, bringing together experts and organizations from around the world. The meeting concluded with the adoption of the Doha Declaration on LM (4); a collective call to action urging the integration of LM into mainstream healthcare to tackle the root causes of NCDs. Building on this momentum, this white paper outlines the challenges surrounding NCDs and justifies why LM should be adopted to address them. It then provides recommendations and future directions for integrating LM into healthcare systems.

### 1.1 Challenges surrounding NCDs: building a case for LM adoption

#### 1.1.1 NCDs are a global concern

NCDs include major global health concerns such as heart disease, stroke, Alzheimer’s disease and other forms of dementia, chronic respiratory disease, cancer, and diabetes. These conditions are widespread, (5) and they are collectively responsible for 70% of deaths (6) and 63% of Disability-Adjusted Life Years (DALYs) (7) worldwide. NCDs share common risk factors such as tobacco use, unhealthy diets, physical inactivity, and harmful alcohol consumption, which are the cornerstones of therapeutic interventions in LM. NCDs result in increased morbidity, functional limitations and disability, an impaired quality of life, and cause premature mortality (8, 9).

NCDs have a profound impact on global economic health, increasing healthcare spending, reducing national income, affecting macroeconomic productivity and contributing to household impoverishment (10–13). Between 2011 and 2030, the cumulative global cost of NCDs is projected to range between USD 30 and 47 trillion (14). The economic burden is particularly severe in low-resource communities, where healthcare costs are often borne directly by patients. The resulting financial strain may lead to catastrophic health expenditures, impoverishment, and cutting back on essential non-medical spending (such as purchasing food). Therefore, families may experience compromised food security, limited access to other basic needs, and a decline in overall well-being (10, 11).

Beyond direct healthcare costs, NCDs exert substantial indirect economic effects. Labor-force participation and productivity are reduced through absenteeism, decreased work capacity, early retirement, and premature mortality. The total burden of care—often shouldered by family members - reduces workforce capacity and suppresses economic output (11). These combined pressures amplify the global economic strain and highlight the need for preventive strategies that can mitigate both the health and financial toll of NCDs.

Complicating this further is that many people may be unaware they are suffering from one or more NCDs (9). Addressing the root causes of NCDs is central to the preventive and therapeutic strategies advocated by LM, which focuses on sustainable behavioral changes to reduce disease burden and improve health outcomes. Given the growing burden of these conditions, implementing effective strategies to prevent their onset and improve their management is a global health priority.

#### 1.1.2 Current evidence for LM in NCD prevention and management

Approximately 80% of all NCDs can be prevented (15), and evidence shows that LM plays a significant role in improving health outcomes (1, 16). For instance, adherence to LM principles can prevent over 80% of heart disease cases and 90% of type 2 diabetes cases, two conditions that together contribute for a large proportion of global morbidity and mortality. Several randomized controlled trials have reported that such interventions can be effective. One trial in patients with coronary artery disease found that those who adopted comprehensive lifestyle changes experienced better cardiovascular outcomes, including slower disease progression, and even measurable reversal – compared with controls (17). The TRIUMPH trial (18) demonstrated that a combination of the DASH diet, caloric restriction, supervised exercise training sessions, and behavioral weight loss counselling achieved a significant reduction of blood pressure in patients with resistant hypertension compared to those receiving standard care. Participants enrolled in the intensive weight-management program of the DiRECT trial were significantly more likely to achieve diabetes remission than those who continued with usual care by the end of the study (19). Other trials have shown that multicomponent LM approaches can help alleviate symptoms of depression (20) and improve sleep quality (21). Beyond these, LM can also contribute to the prevention and management of other chronic conditions, including cancer, obesity, mental health conditions, dementia, and other NCDs (1, 3, 22–26).

Given its central role in NCD prevention, there is a growing emphasis on ensuring that healthcare professionals are trained and competent in LM. Training healthcare professionals in LM offers a dual advantage. First, it equips healthcare professionals with the knowledge and practical skills to motivate patients to adopt and maintain healthier behaviors (27). Further, when healthcare professionals adopt LM principles in their own lives, they not only benefit personally but they are more likely to inspire their patients to consider LM interventions for their health (24).

Together, these examples highlight LM as a cornerstone in NCD prevention and management, whose impact depends on both patient adherence and the ability of healthcare professionals to effectively prescribe and model its principles.

Beyond its clinical effectiveness, LM facilitates patients’ active role in managing their health and promoting healthy behavior change (28). To maximize its long-term impact, LM should be implemented using a life course approach, recognizing that early-life behaviors and family routines play a foundational role in shaping health trajectories and achieving intergenerational health improvements (29). Together, these findings underscore the critical role of LM in transforming healthcare by addressing the root causes of chronic disease and fostering lifelong well-being.

#### 1.1.3 The business case for LM

NCDs account for 90% of healthcare expenditures in the United States (30). Cost analyses indicate that investment in NCD prevention interventions yields favorable returns, with multiple studies showing that policies and programs targeting risk factors can lower health system costs and reduce the economic burden on households (6, 31–33). On a global scale, investments in the prevention and treatment of NCDs between 2015 and 2030 are estimated to prevent 15 million deaths and generate a return on investment (ROI) of 5.6 (6).

Specific examples further illustrate the economic value of LM interventions. In a study of Medicare recipients using a digital lifestyle management program (“Prevent”), the 10-year ROI was estimated at USD 12,600 for individuals with prediabetes and USD 8,900 for those with cardiovascular risk factors (31). In Australia, every AUD invested in structured diet and physical activity interventions for pregnant women during antenatal care yields an estimated AUD 4.75 in savings from reduced adverse pregnancy outcomes (33). Globally, for every USD invested in workplace LM interventions, an estimated return of USD 1.50 is expected within 5 to 6 years (34). Taken together, these findings underscore the cost-effectiveness of LM and highlight its strategic value in strengthening healthcare systems and mitigating the growing burden of NCDs (35–38).

There thus remains a pressing need to evaluate the economic implications of LM. The magnitude of these economic benefits may vary across countries, depending on NCD prevalence, the availability of screening programs, and access to primary, secondary, and tertiary care (32). Research should focus on modeling the potential cost savings of LM interventions compared with conventional medical treatment across different healthcare systems (39). Such evidence could inform health policy decisions and support the broader adoption of LM strategies at both national and international levels.

#### 1.1.4 Patient perspectives on LM interventions

Evaluating patient perspectives on LM interventions is necessary, as their experiences, motivations, and perceived barriers directly influence engagement, adherence, and long-term outcomes. Overall, studies suggest that patients are optimistic and excited about the benefits of LM interventions (40, 41). In a Canadian LM program, participants reported that their overall engagement with the intervention was enhanced by personalized care, the presence of supportive healthcare professionals, and more frequent check-ins (42). Another study involving individuals living with mental illness found that LM principles aligned with their values and were perceived as more effective than pharmacological treatments (43). Nonetheless, the same study also identified important barriers, such as a lack of self-efficacy and the perception that LM practices were too burdensome to implement in daily life (43).

Another example comes from a diabetes prevention program with LM components delivered by the UK’s National Health Service. Prediabetic participants reported engagement, satisfaction, and positive behavioral changes (e.g., increasing daily steps, learning new healthy recipes, and achieving weight loss), while praising the support received from healthcare professionals and peers (44). However, the study also noted challenges, including scheduling issues, large group sizes, and limited resources (44).

Despite these encouraging signs, research capturing patient perspectives after participation in LM programs remains limited. More studies are needed to systematically assess patient experiences across diverse populations and contexts, helping to identify strategies that enhance adherence, improve program design, and maximize long-term effectiveness.

#### 1.1.5 Assimilating LM into the healthcare professions curriculum

Healthcare professionals today feel inadequately prepared to counsel patients on LM, despite recognizing its importance in clinical practice (45). In response, the American College for Preventive Medicine and the American Medical Association have jointly advocated for the integration of all LM pillars into both undergraduate and graduate medical school training, alongside team-based care models involving healthcare professionals across disciplines (45). Evidence suggests that LM education and training not only improve medical students’ knowledge and skills, but also positively influence their personal lifestyles, with 60% reporting improved personal diet and 55% reporting increased exercise (46).

Despite growing evidence of LM’s benefits, most current medical school programs addressing LM remain didactic in nature. Moreover, these learning experiences tend to focus primarily on nutrition and physical activity, with only a quarter of programs incorporating at least four pillars into their teaching sessions (47). Shifting toward more active participation and learning may be more effective in equipping students with practical skills. LM competencies are currently being incorporated into qualifying and licensing examinations for students and healthcare professionals (47, 48).

#### 1.1.6 LM and health disparities

NCDs are disproportionately prevalent among marginalized and underserved communities. (15, 49) Within these populations, NCDs are often diagnosed at more advanced stages, leading to worse health outcomes and incurring higher management costs (49). One of the key factors shaping these health inequities is the social determinants of health (SDoH) — defined as the conditions in which people are born, grow, live, work and age, and people’s access to power, money and resources (50). The structural and environmental conditions of marginalized and underserved communities shape daily opportunities and constraints, thereby influencing an individual’s ability to engage in healthy behaviors. The significance of SDoH is underscored by evidence showing that nonclinical factors (i.e., behaviors, social circumstances, and environmental exposures) are responsible for a greater share of premature mortality than medical care itself (51). For this reason, systematic assessment of SDoH should be integrated into standard healthcare visits, rather than being viewed as optional. (52) Targeting SDoH is therefore central to reducing health disparities, strengthening prevention efforts, and improving population health (49).

Recognizing this, efforts have increasingly focused on developing LM programs that directly target SDoH and improve equity in access to care. For example, in Washington, DC, a pilot program aimed at addressing food insecurity and nutritional education by providing wholesome food during pregnancy and up to two years postpartum, while also encouraging antenatal and well-child checkups (52). In parallel, The American College of Lifestyle Medicine launched the Health Equity for Lifestyle Medicine (HEAL) Initiative, which aims to address health disparities by delivering on the promise of LM (53). Through this initiative, efforts have focused on creating community engagement around the challenges surrounding systemic barriers and demonstrating how LM helped overcome them (53).

LM may be particularly relevant in low- and middle-income countries, where the burden of NCDs is especially high (22). These countries account for more than three-quarters of global NCD-related deaths each year, highlighting an urgent need for accessible and preventive strategies (15). In this context, LM can be adapted to local realities through programs delivered by trained community health workers and peer educators (54–56). Such programs can help ensure that interventions are culturally appropriate and aligned with local practices and beliefs. By building on existing community structures, these approaches remain feasible even in resource-constrained environments and offer a scalable model that reduces reliance on costly treatments (54–56).

### 1.2 Recommendations and future directions

The Doha Declaration on LM emphasizes a holistic approach to preventing, managing, and reversing NCDs. In line with its call for evidence-based implementation, research, and education, we outline four key areas of action to embed LM within society: (1) incorporating LM into the current healthcare system, (2) addressing limited awareness and education, (3) addressing the lack of resources, and (4) identifying research gaps. The following subsections discuss each of these areas in detail.

#### 1.2.1 Incorporating LM into the current healthcare system

One way to embed LM within society is through its systematic incorporation into existing healthcare systems. However, training in this area remains limited and many healthcare professionals continue to prioritize surgical or pharmacological interventions over LM, despite convincing evidence in favor of the latter (57). Even when healthcare professionals attempt to integrate LM into their practice, they are often insufficiently equipped to do so (57). As a result, clinicians often rely exclusively on pharmacological or surgical interventions. It remains imperative to ensure that healthcare professionals receive appropriate LM training to shift the focus of care from disease management to primary prevention (Table 1). To address this gap, important steps in this direction have already been taken, including the development of LM physician and practitioner competencies, board certification, and continuing medical and professional education programs (58). The Lifestyle Medicine Residency Curriculum further provides a structured pathway that combines educational and practical components, enabling medical residents to incorporate LM into their professional and personal lives. (5) In parallel, core competencies, clinical guidelines and protocols, and performance measures have been developed to codify LM in healthcare (58–63).

**Table 1:** Challenges and solutions to advance LM.

Area of action	Current challenge	Proposed solution
1. Incorporating LM into the current healthcare system	Gaps in healthcare provider knowledge	Provide appropriate training for healthcare professionals to counsel patients effectively. Implement LM competencies into residency programs. Promote uptake of LM board certification and continued medical/professional education.
2. Improving LM awareness among the general public	Limited awareness of LM among the general public	Engage community health workers in outreach programs. Collaborate with cultural and religious leaders. Establish LM centers staffed by multidisciplinary teams. Use motivational interviewing to encourage behavior change.
3. Addressing lack of resources	Lack of resources Healthcare workforce disparities	Advocate for the incorporation of LM practices into healthcare systems to improve health outcomes. Promote LM to reduce healthcare professional burnout and increase job satisfaction. Use digital and virtual technologies to disseminate LM knowledge.
4. Identifying research gaps on SDoH and health outcomes	Lack of data Poorly designed studies with small sample sizes	Design large scale, longitudinal studies to assess the complex interactions between LM components and health outcomes Adopt a systems-level approach

NCDs lead to a wide range of functional limitations and disabilities, undermine mental health, and heighten pain and suffering, collectively resulting in a markedly reduced quality of life (8, 9). Quality of life, as defined by the World Health Organization (WHO), refers to a person’s “perception of their position in life in the context of the culture and value systems in which they live and in relation to their goals, expectations, standards and concerns” (64). Moreover, quality of life has taken on increasing significance in recent decades as rising life expectancy - driven by advances in medicine and public health - has shifted attention toward not just living longer but living well. Validated tools such as the WHO’s WHOQOL (65), the Centers for Disease Control and Prevention’s HRQOL (66), or the Significant Quality of Life Measure (SigQOLM-36) (67) therefore play a vital role in evaluating quality of life outcomes in LM and should be fully integrated into healthcare systems.

Building on these foundational efforts, further action is needed to expand the reach and impact of LM, particularly through institutional and environmental change. To this end, we propose a set of strategies aimed at promoting sustainable healthcare delivery.

Healthcare institutions can serve as focal points for initiatives that enhance environmental health and the community. In this context, LM can contribute to environmental sustainability by encouraging healthcare practices that minimize environmental impact. Clinical care consumes substantial resources and generates harmful air pollutants, greenhouse gases, and waste (68). Where possible, environmental impacts and resource stewardship should be considered when selecting one of several possible interventions. For instance, healthcare institutions can opt for intravenously-administered anesthetics to reduce potent greenhouse gas emissions, reduce unnecessary testing and over-prescription of drugs, opt for laparoscopic surgeries where possible, recommend green or social prescriptions to patients as appropriate, and advocate for healthier policies to benefit communities (68–70).

While institutional change is essential, equal attention must be given to how these strategies are implemented (Table 1). Without institutional alignment, even well-trained professionals may struggle to implement LM consistently in their practice. Embedding LM into healthcare systems therefore requires not only preparing clinicians but also creating structures that enable them to prioritize lifestyle interventions and measure their impact. By aligning education, clinical practice, and institutional policies, LM can become an integral component of routine healthcare rather than an optional addition.

#### 1.2.2 Improving LM awareness among the general public

Public awareness of the efficacy of LM remains limited, particularly in relation to its role in the prevention and management of NCDs. This challenge is further exacerbated in settings with low health literacy, where individuals often struggle to access, interpret, and apply reliable health information (54). Ensuring culturally sensitive LM programs is especially important in religiously- and culturally- diverse contexts, where LM principles can be adapted to align with local food practices and preparation techniques (71, 72). Adopting patient-centered and culturally-sensitive approaches can strengthen the relationship between patients and providers and enhance commitment to behavior change (73) and can further strengthen collective shifts toward healthier lifestyles (74).

Examples of successful community-driven interventions demonstrate the potential of holistic LM approaches. Repurposing community health workers and other healthcare professionals to engage in outreach programs has proved beneficial for raising awareness of LM and improving engagement in preventive health behaviors in some settings (54). Underserved communities experience better health outcomes and higher patient satisfaction when cared for by a diverse health workforce, underscoring the importance of advancing minority practitioner representation and education (52, 75). An initiative aimed at educating young mothers, delivered by other trained mothers, resulted in an overall improvement in both dietary habits and physical activity among children (76). LM education programs, delivered by trained volunteers and medical students, have equipped middle and high school students with the ability to make healthier lifestyle choices (77). Comparable success has been observed in workplace programs targeting adults, where culturally adapted LM interventions embedded in daily professional routines helped normalize healthy behaviors (74).

Another proposed model involves the development of community-based LM centers staffed by multidisciplinary teams, including nutritionists, exercise physiotherapists, psychologists, and other allied health professionals (78). These centers could deliver tailored nutrition and physical activity prescriptions of diverse patient groups, facilitate access to community resources, strengthen peer support networks, and offer referrals to healthcare clinics (78). By integrating LM within a broader system of care, these multisectoral partnerships and initiatives not only enhance continuity but also position lifestyle interventions as integral to addressing SDoH, reducing health inequities and reversing the rising trend of NCDs.

While community-based strategies demonstrate considerable promise to increase LM awareness among the general public, equal attention must be given to how such strategies are implemented (Table 1). The historical dominance of conventional approaches in medical practices has often overshadowed LM in the public sphere, limiting its visibility and perceived legitimacy (79). To overcome these barriers, dissemination must extend beyond simple awareness campaigns to include transparent communication of evidence, active involvement of community stakeholders, and integration of LM into trusted social and cultural networks. Such approaches can help increase acceptance and build the trust necessary for LM to become embedded within daily life.

#### 1.2.3 Addressing lack of resources

Healthcare professionals frequently report a lack of time to address patient needs and express concern that incorporating LM could exacerbate backlogs and delays (57). Further, a well-equipped and highly trained health workforce will migrate to urban settings, depriving marginalized and rural residents of quality healthcare (54). However, advocating for an LM approach can diminish overall demand and costs (57). Evidence suggests that healthcare professionals who practice LM report lower levels of burnout, greater meaning in their work, and higher personal and professional satisfaction compared to those who do not, factors that can enhance healthcare worker retention rates (80). Additionally, digital and virtual tools can also play a key role by making LM knowledge more accessible the lay public (54). Human, financial, and organizational resources must be allocated towards identifying and addressing gaps in healthcare and understanding a hierarchy of how LM can be seamlessly incorporated (Table 1).

#### 1.2.4 Identifying research gaps on SDoH and health outcomes

Currently, the interplay between SDoH and health outcomes remains insufficiently delineated, primarily due to a lack of data and the complexity of underlying interactions. (81, 82) This lack of clarity is reflected across several domains of research. For instance, although the link between high intake of added sugars and dental caries is well established, further research is required to determine whether a safe level of added sugar exists (83). In addition, the roles of tobacco use and the presence of metabolic syndrome in the prognosis and management of periodontal disease remain poorly understood and deserve further investigation (83). Knowledge gaps persist regarding the type and dosage of physical activity: While aerobic, resistance, balance, and flexibility training are all recognized components of physical health, the specific dose–response relationships between these modalities and health outcomes remain poorly understood (82). Conditions such as acne, rosacea, and skin cancer may be influenced by lifestyle behaviors (84). The role of nutrition in cognitive and mood disorders remains insufficiently understood, and additional research is needed to elucidate the mechanisms through which dietary patterns influence psychological well-being (82).

Although closing these gaps is a priority, it is equally important to recognize that strategies may succeed in some contexts but fail in others. For example, approaches that prove effective in a well-resourced urban hospital may be unrealistic in rural clinics with limited staff, equipment, or funding. Because SDoH strongly influence people’s ability to adopt and sustain healthy behaviors, adopting a systems-level approach to examine how these determinants shape health outcomes is essential (81). Such an approach can guide the design of solutions that are sensitive to the diverse environments in which interventions are applied. In this way, LM strategies can be ensured to remain evidence-based, equitable, and sustainable across different populations and healthcare settings.

## 2 Limitations

One of the assumptions of LM is that patients are able to understand its principles and utility, and apply them in their daily lives. However, patients may not be able to do so due to poor health literacy. Policies to boost health literacy and self-management skills can contribute to lower NCD burden (85). The evidence base for the effectiveness of LM interventions remains underexplored. Comprehensive monitoring and evaluation programs are needed to assess outcomes, refine approaches, and build a stronger case for appropriate, optimum adoption of LM (86). While LM research outcomes often focus on a single disease, patients tend to present with multiple NCDs. These diseases are multifactorial and influenced by SDoH, complicating the clinical picture and disease management. Further research and frameworks will, no doubt, aid and support patients with multiple comorbidities and complex clinical presentations (87).

## 3 Conclusion

This white paper, building on the recommendations outlined in the Doha Declaration on LM, highlights LM as a cornerstone for the prevention and management of NCDs, extending its impact beyond clinical settings into communities and society (88). By addressing the root causes of NCDs, LM can reduce prevalence of NCDs and their complications, lower healthcare costs, enhance workplace productivity, and promote overall well-being. As evidence continues to illuminate and highlight the complex interplay between lifestyle factors and health outcomes, healthcare systems should place increasing emphasis on LM initiatives as a strategic priority. To maximize its impact, LM strategies must be culturally responsive and adapted to the needs of diverse populations, including marginalized groups and individuals already living with NCDs. The future we envision is one in which, by harnessing the potential of LM, individuals are empowered with knowledge and skills to manage their health, resulting in improved well-being, lower healthcare costs and a healthier society overall.

## Data Availability

The original contributions presented in the study are included in the article/supplementary material, further inquiries can be directed to the corresponding author.
